# Frailty Index and functional level upon admission predict hospital outcomes: an interRAI-based cohort study of older patients in post-acute care hospitals

**DOI:** 10.1186/s12877-020-01550-7

**Published:** 2020-05-05

**Authors:** Hanna Kerminen, Heini Huhtala, Pirkko Jäntti, Jaakko Valvanne, Esa Jämsen

**Affiliations:** 1 Faculty of Medicine and Health Technology, and the Gerontology Research Centre (GEREC), Tampere University, P.O. Box 100, 33014 Tampere, Finland; 2 Centre of Geriatrics, Tampere University Hospital, Central Hospital, P.O. Box 2000, 33521 Tampere, Finland; 3 Faculty of Social Sciences, Tampere University, P.O. Box 100, 33014 Tampere, Finland

**Keywords:** Older people, Aged, Geriatric assessment, Functional ability, Frailty, Frailty index, Inpatients, Post-acute care, Hospital outcomes

## Abstract

**Background:**

Geriatric assessment upon admission may reveal factors that contribute to adverse outcomes in hospitalized older patients. The purposes of this study were to derive a Frailty Index (FI-PAC) from the interRAI Post-Acute Care instrument (interRAI-PAC) and to analyse the predictive ability of the FI-PAC and interRAI scales for hospital outcomes.

**Methods:**

This retrospective cohort study was conducted by combining patient data from interRAI-PAC with discharge records from two post-acute care hospitals. The FI-PAC was derived from 57 variables that fulfilled the Frailty Index criteria. Associations of the FI-PAC and interRAI-PAC scales (ADLH for activities of daily living, CPS for cognition, DRS for mood, and CHESS for stability of health status) with hospital outcomes (prolonged hospital stay ≥90 days, emergency department admission during the stay, and in-hospital mortality) were analysed using logistic regression and ROC curves.

**Results:**

The cohort included 2188 patients (mean age (SD) 84.7 (6.3) years) who were hospitalized in two post-acute care hospitals. Most patients (*n* = 1691, 77%) were discharged and sent home. Their median length of stay was 35 days (interquartile range 18–87 days), and 409 patients (24%) had a prolonged hospital stay. During their stay, 204 patients (9%) were admitted to the emergency department and 231 patients (11%) died. The FI-PAC was normally distributed (mean (SD) 0.34 (0.15)). Each increase of 0.1 point in the FI-PAC increased the likelihood of prolonged hospital stay (odds ratio [95% CI] 1.91 [1.73─2.09]), emergency admission (1.24 [1.11─1.37]), and in-hospital death (1.82 [1.63─2.03]). The best instruments for predicting prolonged hospital stay and in-hospital mortality were the FI-PAC and the ADLH scale (AUC 0.75 vs 0.72 and 0.73 vs 0.73, respectively). There were no differences in the predictive abilities of interRAI scales and the FI-PAC for emergency department admission.

**Conclusions:**

The Frailty Index derived from interRAI-PAC predicts adverse hospital outcomes. Its predictive ability was similar to that of the ADLH scale, whereas other interRAI-PAC scales had less predictive value. In clinical practice, assessment of functional ability is a simple way to assess a patient’s prognosis.

## Background

Geriatric syndromes are common clinical conditions in older adults [1]. They are often connected to each other with multiple shared underlying aetiological factors that involve different organ systems [1]. Frailty is a geriatric syndrome in which the patient’s ability to resist stressful events is reduced as a result of age-related cumulative decline in many physiological systems [2]. At least in its early stages, frailty is a potentially reversible condition [3].

Frail older patients [4, 5] and those suffering from other geriatric syndromes [6, 7] are vulnerable to adverse outcomes. Frailty predicts prolonged hospital stay [8–10] and in-hospital mortality [10–12]. Impaired functional ability in activities of daily living (ADLs) and impaired cognition predict all-cause mortality among hospitalized patients [13, 14]. Symptoms of depression associate with in-hospital mortality, all-cause mortality, and length of hospital stay [15, 16]. In addition, stability in health state, measured by combining different instability symptoms with functional ability, declined cognition, and poor prognosis, predicts all-cause mortality among institutionalized patients and patients with neurological conditions [17, 18], but studies among hospitalized patients are lacking.

Even though geriatric syndromes are highly prevalent among acutely ill hospitalized patients [6, 19], the recognition rate of these conditions is low [6]. However, hospitalization offers opportunities to identify and act on geriatric syndromes and undiagnosed diseases [20]. The Comprehensive Geriatric Assessment (CGA) was developed to improve the identification of older patients with geriatric syndromes [19]. The CGA includes an assessment of the patient’s medical, psychological, cognitive and functional problems, as well as environmental and social factors. The assessment leads to a treatment plan, rehabilitation, and follow-up [19]. Performing the CGA during a stay in acute care increases the patient’s likelihood of being alive and living at home one year later [19].

There is currently no clear consensus about the contents of the CGA, and several different CGA approaches have been developed. One example is the interRAI assessment system, which can be used as a CGA tool [21]. Similarly, frailty does not yet have an internationally recognized standard definition, nor is there a gold standard for detecting it [22]. Instead, there are multiple frailty instruments that are based on one of two widely used frailty models: the phenotypic model [23] and the cumulative deficit model [24]. The phenotypic model defines frailty as the presence of three or more of five factors in an individual [23]. In the cumulative deficit model, frailty is defined as the cumulative effect of individual deficits [24]. The Frailty Index is based on this latter model [24]. Although the interRAI instrument is lacking a frailty scale, it can be derived from the database [25].

To the best of our knowledge, no previous studies have dealt with the prognostic effects of the Frailty Index and different interRAI scales in post-acute care. The aims of this study were 1) to derive a Frailty Index (FI-PAC) from the interRAI Post-Acute Care instrument (interRAI-PAC), 2) to determine how the FI-PAC associates with hospital outcomes (in-hospital mortality, prolonged hospital stay, and emergency department admission), and 3) to clarify how the other scales of the interRAI-PAC compare in the prediction of hospital outcomes.

## Methods

### Design and setting of the study

**Fig. 1:**
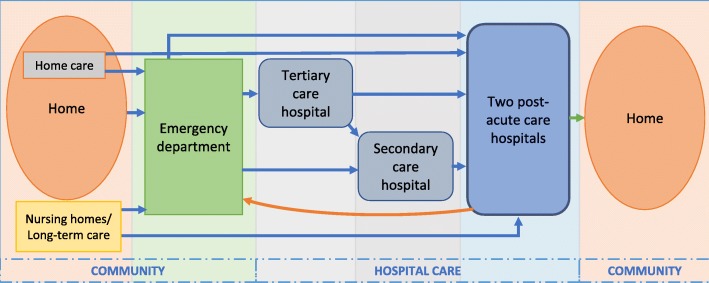
Illustration of the organizational structure of geriatric care in the city of Tampere, Finland, and the movement of patient-flow through care settings (*blue arrows* from home to hospital, *orange arrow* emergency department admission during the stay in post-acute care hospital, *green arrow* from the post-acute care hospital to home)

This study was a retrospective cohort study among patients aged 70 and older who were hospitalized in two geriatric post-acute care hospitals in Tampere (population base 232,000, of which 11% is aged 70 years or older), Finland, during the period of 1 February 2013 to 31 May 2016. These hospitals (230 and 190 beds) offered subacute care and rehabilitation for older patients who were first hospitalized in a tertiary or secondary care hospital (Fig. 1). In addition, one of the hospitals served as a supporting hospital for home care clients. Consequently, home care nurses or physicians in the emergency room could refer these patients directly to this hospital without hospitalization in an acute care setting. At the end of 2015, this hospital was closed due to organizational changes.

**Fig. 2:**
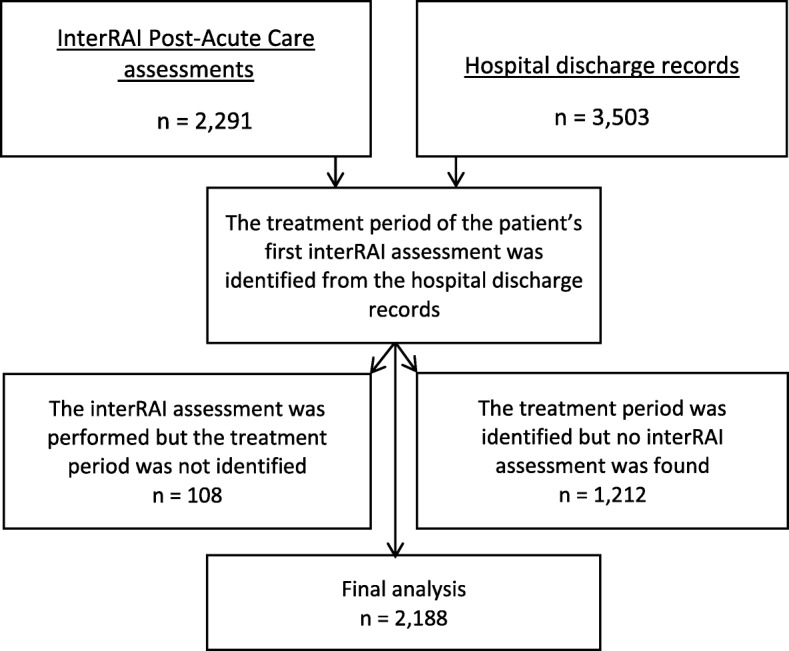
Formation of materials

The results of the interRAI-PAC assessments (see below) were linked to hospital discharge records, which contained information about the patient’s usual residence, the place he/she was admitted from, dates of admission and discharge, discharge diagnosis and destination, and, when applicable, death during hospitalization. In patients with several hospitalizations during the observation period, the first to which interRAI data could be linked was included in this study. Information on the patient’s chronic diseases, functional ability, previous falls, smoking habits, and Body Mass Index (BMI) were collected from the interRAI-PAC. Some 2188 patients were included in the final analysis (Fig. 2).

### InterRAI Post-Acute care instrument (interRAI-PAC)

There are several interRAI instruments with similar core items and divergent instrument-specific domains. The interRAI-PAC is designed for post-acute care and rehabilitation settings [26]. It contains information across domains, including functioning on the physical, cognitive and psycho-social levels as well as sociodemographic data, medical diagnoses, and current symptoms. Single items are combined to compose validated scales that measure different aspects of functional ability. InterRAI instruments have substantial interrater reliability [27, 28].

The use of interRAI-PAC instrument was started on 1 February 2013 in one post-acute care hospital and gradually in the other hospital. All the wards in this particular hospital had started to use interRAI-PAC by the beginning of the year 2016. Trained nurses performed the assessment within a few days of the patient’s admission to the ward. To obtain relevant information, they interviewed the patient and family members, observed the patient, and reviewed the medical records. The assessment consisted of 150 variables. The only missing variables were for weight or height (in 23 patients).

Based on previous findings of prognostic factors related to the outcomes of older inpatients [4–7, 10, 13, 16, 18], associations of the interRAI scales measuring cognitive functions, ADLs, mood, and stability of health state were used in this study. The Cognitive Performance Scale (CPS) describes the cognitive status of the patient based on an algorithm [29]*.* The Activities of Daily Living Hierarchy Scale (ADLH) is an algorithm that considers a measure of ADL performance in locomotion, eating, toilet use, and personal hygiene [30]. The Depression Rating Scale (DRS) is based on existing symptoms of depression [31]. The Changes in Health, End-stage disease, and Signs and Symptoms Scale (CHESS) is a summary measure based on decline in cognition and ADL performance, certain symptoms (for example, weight loss, shortness of breath, and oedema), and ratings of a prognosis of less than six months, and it is designed to identify individuals at high risk for clinically significant decline [17].

### Derivation of the Frailty Index from the interRAI-PAC instrument

The Frailty Index is a method to measure frailty in relation to the accumulation of health deficits [32], and it can be calculated from a variety of databases according to the standard procedure for selecting individual deficits [32]. The Frailty Index is the proportion of deficits present in an individual out of the total number of variables considered [32], and so higher scores are associated with adverse hospital outcomes – for example longer length of hospital stay, new discharge to a nursing home, and death [9, 10]. The Frailty Index from the interRAI Acute Care instrument (FI-AC) was previously derived and published by Hubbard et al. in 2015 [25]. The interRAI-AC instrument includes the same core items as the interRAI-PAC but has fewer items in total.

In this study, the Frailty Index (FI-PAC) was derived from the interRAI-PAC according to the standard procedure and the well-defined criteria created by Searle et al. [32], and leaning on the coding of variables in FI-AC. In short, all the items of the interRAI-PAC were evaluated against the Frailty Index criteria independently by two geriatricians. Secondly, eventual differences were negotiated to achieve a consensus of appropriate variables in post-acute care patient population. Finally, variables were compared with the coding of FI-AC [25]. There are several explanations for the differences between FI-PAC and FI-AC. First, some variables that were used in FI-PAC are not recorded in interRAI AC. Second, some differences are based on the differences in interpretation of the criteria for selecting appropriate variables to FI, mainly based on different characteristics of patient populations in post-acute and acute care settings. Finally, the Depression Rating Scale, Pain Scale, and Aggressive Behaviour Scale were included in the FI-PAC instead of using single variables, because the scales reflect both the patient’s situation and criteria for selecting variables to FI better than separate variables related to the issue. Of the variables considered, 57 variables were chosen for the FI-PAC [Additional file 1]. The FI-PAC was calculated for each patient by summing deficit points and dividing the sum by the total number of deficits considered. The only missing item was BMI (in 23 patients), and the denominator was adjusted to 56 items for these patients.

### Outcome measures

*Prolonged hospital stay.* Length of hospital stay was determined as the difference between the date of admission and the date of discharge. Length of stay in post-acute care hospital was recorded only for the patients who were discharged to their usual residency (own home or nursing home). It was not recorded for the patients who had emergency department admissions or who died during the hospital stay. In addition, length of hospital stay was not recorded for the patients who were admitted from home but were discharged to nursing home for long-term care (*n* = 69). This is because the delay of a new nursing home placement was most probably more dependent on the organizational factors than on patient’s condition. Length of hospital stay was dichotomously classified as less than 90 days and 90 days or more according to the usual cut-off for long-term care [33]. Hospitalization for 90 days or more was defined as a prolonged hospital stay.

*Emergency department admission* was recorded for the patients who were transferred to the emergency department during their post-acute care treatment period.

*In-hospital mortality* was recorded from the discharge records and defined as death during the stay in the post-acute care hospital. In addition, deaths in patients who were referred to an acute care hospital because of an acute illness and who died there on the same day were also counted as in-hospital deaths (*n* = 4).

### Statistical analysis

Patient characteristics were described using frequencies and percentages. The distribution of the FI-PAC was tested in all patients as well as in sex and age groups; the results are presented as means and standard deviations. The predictive ability of the FI-PAC on outcome measures was investigated using binary logistic regression analysis, adjusted for age and sex. Logistic regression analyses were also performed for sex and age subgroups. The receiver operating characteristic curve (ROC) and the area under the curve (AUC) with 95% confidence intervals (CIs) were calculated to clarify the discriminative ability of the FI-PAC for hospital outcomes. For each outcome measure, the optimal cut-off point of the FI-PAC for sensitivity and specificity was calculated using the Youden method, and positive and negative predictive values (PPV and NPV) were determined. To compare the predictive ability of the FI-PAC to that of existing interRAI scales, the ROC curve and the AUC with corresponding 95% CIs for hospital outcomes were also calculated for the ADLH, CHESS, CPS, and DRS scales. Data management and analysis were performed using IBM SPSS Statistics version 25.

### Ethics

Retrospective register-based studies in which the subjects are not contacted are not considered medical research by Finnish legislation (Medical Research Act 1999/488 § 2) [34] and, therefore, ethics committee approval was not required. Retrospectively collected health register data could be used for this study with permission of register owner without participants’ informed consent, based on current legislation (Data Protection Act 2018/2010, Act on the Publicity of Official Documents 1999/621, European Union General Data Protection Regulation) [35–37]. Research plan was institutionally reviewed and permission to use the interRAI-PAC assessments and hospital discharge records was hence obtained from the administration of the City of Tampere (decision the Director of Hospital Services, in August 30, 2016).

## Results

### Characteristics of the patients

The cohort included 2188 patients with a mean age (SD) of 84.7 (6.3) years. Most of the patients were female (*n* = 1499, 69%) (Table 1). Almost half of the patients (46%, *n* = 1004) had a memory disorder diagnosis. Only 12% of the patients (*n* = 255) were independent in all basic activities of daily living (BADLs) (bathing, personal hygiene, dressing, walking, locomotion, transfer to toilet, toilet use, bed mobility, and eating), while 18% (*n* = 395) were totally dependent on caregivers for all BADLs. Half of the patients came to hospital straight from home and the other half came from an acute care hospital.

**Table 1:** Baseline characteristics and outcomes of the patients (*n* = 2188)

	n	%
Female	1499	68.5
Age (years)		
70–79.9	498	22.8
80–89.9	1234	56.4
≥ 90	456	20.8
Age (years) mean (SD)	84.7	(6.3)
Usual residence		
Own home	1959	89.5
Nursing home/long-term care	229	10.5
Chronic diseases		
Alzheimer’s disease	737	33.7
Other memory disorder	217	9.9
Alzheimer’s disease and other memory disorder	50	2.3
Congestive heart failure	685	31.3
Coronary heart disease	572	26.1
Diabetes	528	24.1
Cancer	325	14.9
Stroke/cerebrovascular accident	228	10.4
Depression	209	9.6
COPD	156	7.1
Parkinson’s disease	59	2.7
Independent in Activities of Daily Living		
Bathing	316	14.4
Personal hygiene	572	26.1
Dressing	649	29.7
Toilet use	859	39.3
Transfer to toilet	1003	47.5
Walking	1014	46.3
Bed mobility	1039	47.5
Eating	1726	78.9
Primary mode of locomotion at the hospital		
Walking, no assistive device	245	11.2
Walking, with assistive device	1328	60.7
Wheelchair	329	15.0
Bedridden	286	13.1
Falls		
No falls in last 3 months	1077	49.2
Fall(s) 1 to 3 months ago	265	12.1
Fall(s) in last month	846	38.7
Smokes tobacco daily	84	3.8
< 18.5	192	8.9
18.5–24.9	997	46.1
25–29.9	606	28.0
≥ 30	370	17.1
Body Mass Index (BMI) kg/m^2 a^ mean (SD)	25.04	(5.4)
Admitted from		
Home	1028	47.0
Nursing home/long-term care	49	2.2
Acute care hospital	1111	50.8
Ten most common main hospital discharge diagnosis code groups (ICD-10)		
Diseases of the circulatory system (I)	496	22.7
Diseases of the nervous system (G)	408	18.6
Injury, poisoning and certain other consequences of external causes (S or T)	315	14.4
Mental and behavioural disorders (F)	237	10.8
Neoplasms or diseases of the blood (C or D)	129	5.9
Diseases of the musculoskeletal system and connective tissue (M)	128	5.9
Diseases of the respiratory system (J)	110	5.0
Diseases of the genitourinary system (N)	100	4.6
Symptoms and signs, not elsewhere classified (R)	79	3.6
Endocrine, nutritional and metabolic diseases (E)	69	3.2
Outcomes		
Prolonged hospital stay^b^ (*n* = 1691)	409	24.2
Emergency department admission	204	9.3
In-hospital death	231	10.6

^a^ BMI missing, *n* = 23

^b^ In patients who were discharged to their usual place of residence (home or nursing home)

Most of the patients (*n* = 1691, 77%) were discharged to their usual place of residence (own home or nursing home) (Table 1). The median length of stay in post-acute care was 35 days (interquartile range 18–87 days), and 409/1691 patients (24%) had a prolonged hospital stay. Some 204/2188 patients (9%) were admitted to the emergency department. The in-hospital mortality rate was 11% (*n* = 231/2188).

### Distribution of the FI-PAC

**Fig. 3:**
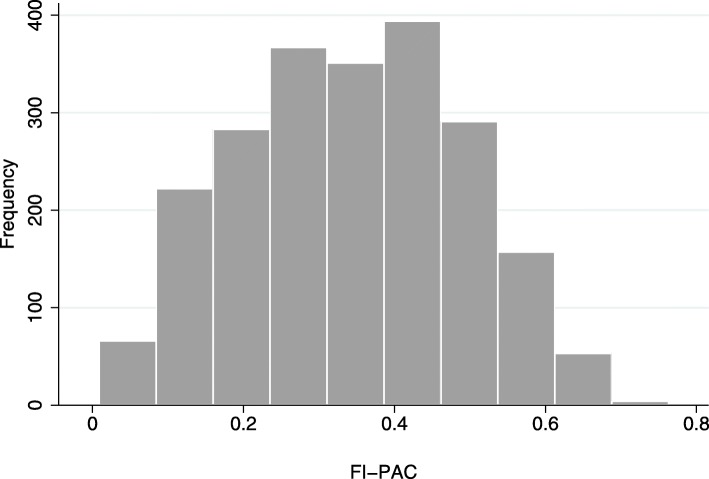
Distribution of the Frailty Index for Post-Acute Care (FI-PAC) among 2188 patients aged ≥70 years in two post-acute care hospitals

The FI-PAC was normally distributed, with a mean (SD) score of 0.34 (0.15), a minimum of 0.01 and a maximum of 0.76 (Fig. 3). There were no significant differences between age and sex groups.

### Association of the FI-PAC and the interRAI scales with hospital outcomes

#### The FI-PAC

In logistic regression analyses adjusted for age and sex, the FI-PAC was associated with prolonged hospital stay, emergency department admission, and in-hospital mortality (Table 2). Each 0.1-point increase in the FI-PAC raised the likelihood of prolonged hospital stay by 91%, emergency admission by 24%, and in-hospital death by 82%. The predictive ability of the FI-PAC to discriminate between patients who did or did not experience an adverse outcome was the best for prolonged hospital stay (AUC 0.75). The predictive ability was lowest for emergency department admission (AUC 0.59). There were no differences between sex and age groups for the ability of the FI-PAC to predict hospital outcomes.

**Table 2:** Discriminative and predictive capacity of the FI-PAC for hospital outcomes

Outcome	OR^a^/0.1 FI increment				Optimal	Sensitivity		Specificity		PPV^**b**^		NPV^**c**^	
		(95% CI)	AUC	(95% CI)	cut-off point	n	(%)	n	(%)	n	(%)	n	(%)
Prolonged hospital stay	1.91	(1.73─2.09)	0.75	(0.72─0.77)	≥0.32	332/409	(81.2)	778/1282	(60.7)	332/836	(39.7)	778/855	(91.0)
Emergency department admission	1.24	(1.11─1.37)	0.59	(0.55─0.63)	≥0.30	148/204	(72.5)	745/1691	(44.1)	148/1094	(13.5)	745/801	(93.0)
In-hospital mortality	1.82	(1.63─2.03)	0.73	(0.70─0.76)	≥0.35	188/231	(81.4)	1057/1957	(54.0)	188/1088	(17.3)	1057/1100	(96.0)

^a^ Adjusted for age and gender

^b^ Positive predictive value

^c^ Negative predictive value

Table 2 shows the sensitivity, specificity, PPV, and NPV of the FI-PAC for each outcome measure. The cut-off point for optimal sensitivity and specificity differed slightly between the outcomes (0.32 for prolonged hospital stay, 0.30 for emergency department admission, and 0.35 for in-hospital mortality). At these optimal cut-off points, sensitivity was higher than specificity. The FI-PAC was equally sensitive in predicting prolonged hospital stay and in-hospital mortality (sensitivity 81%), whereas the sensitivity for emergency department admission was poorer (73%). The specificity was the highest for prolonged hospital stay (61%) and the lowest for emergency department admission (44%). PPV varied from 14% for emergency department admission to 40% for prolonged hospital stay with consistently high NPVs (91–96%). When the cut-off point was elevated to 0.40, which is the usual cut off for frailty [10, 24, 38], specificity rose at the cost of sensitivity (Table 3).

**Table 3:** Predictive capacity of the FI-PAC for hospital outcomes in different Frailty Index (FI) cut-off points

Outcome	FI cut-off point	Sensitivity		Specificity		PPV^**a**^		NPV^**b**^	
		n	(%)	n	(%)	n	(%)	n	(%)
Prolonged hospital stay (≥90 days)	≥0.40	227/409	(56)	975/1282	(76)	227/534	[43]	975/1157	(84)
	≥0.32	332/409	(81)	778/1282	(61)	332/836	[40]	778/855	(91)
Emergency department admission	≥0.40	79/204	[41]	1157/1691	(68)	79/613	[13]	1157/1282	(90)
	≥0.30	148/204	(73)	745/1691	[44]	148/1094	[14]	745/801	(93)
In-hospital mortality	≥0.40	156/231	(68)	1316/1957	(67)	156/797	[20]	1316/1391	(95)
	≥0.35	188/231	(81)	1057/1957	(54)	188/1088	[17]	1057/1100	(96)

^a^ Positive predictive value

^b^ Negative predictive value

#### The interRAI scales (ADLH, CHESS, CPS, and DRS) compared to the FI-PAC

**Fig. 4:**
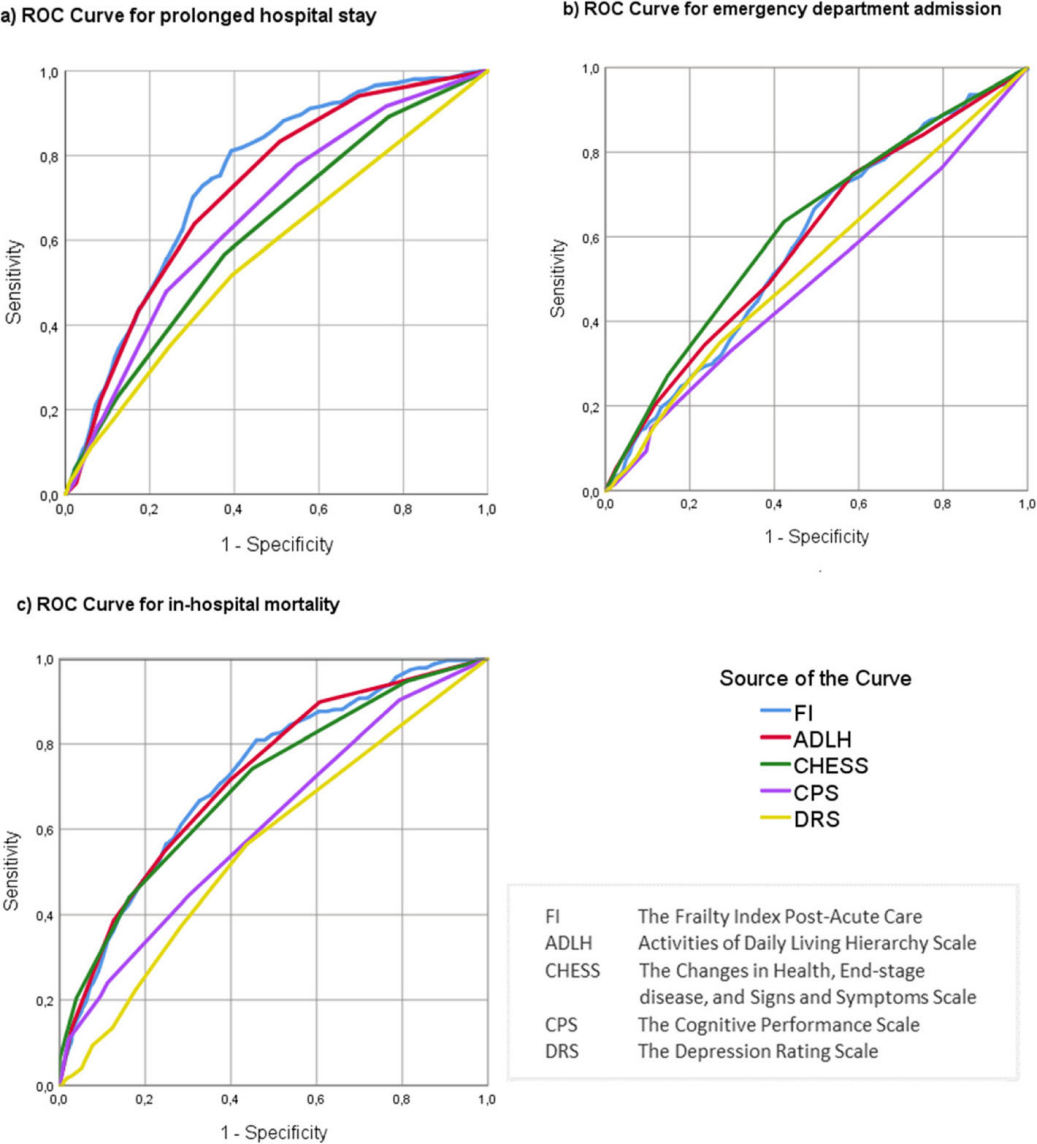
Discriminative ability of the Frailty Index for Post-Acute Care (FI-PAC) and interRAI scales for predicting hospital outcomes among 2188 patients aged ≥70 years in post-acute care hospitals

In a comparison of the interRAI scales and the FI-PAC, the best scales for predicting prolonged hospital stay were the FI-PAC and ADLH with equal discriminative capacity (Table 4 and Fig. 4), and they were also significantly better than CHESS, CPS, and DRS. There were no differences in the predictive abilities of interRAI scales and the FI-PAC for emergency department admission. The best scales for predicting in-hospital mortality were the FI-PAC, ADLH, and CHESS.

**Table 4:** Predictive ability of different interRAI scales compared to the FI-PAC for different hospital outcomes

Scale		Outcome					
		Prolonged hospital stay		Emergency department admission		In-hospital mortality	
**Name**		**AUC**	**(95% CI)**	**AUC**	**(95% CI)**	**AUC**	**(95% CI)**
Frailty Index for Post-Acute Care	FI-PAC	0.75	(0.72─0.77)	0.59	(0.55─0.63)	0.73	(0.70─0.76)
Activities of Daily Living Hierarchy Scale	ADLH	0.72	(0.69─0.75)	0.59	(0.55─0.63)	0.73	(0.69─0.76)
Cognitive Performance Scale	CPS	0.66	(0.63─0.69)	0.50	(0.46─0.58)	0.62	(0.58─0.66)
Depression Rating Scale	DRS	0.57	(0.54─0.60)	0.54	(0.50─0.58)	0.56	(0.52─0.60)
Changes in Health, End-stage disease, and Signs and Symptoms Scale	CHESS	0.62	(0.59─0.65)	0.62	(0.58─0.66)	0.71	(0.67─0.75)

Finally, we repeated the analyses concerning the FI-PAC with the cut-off point < 0.40 vs ≥0.40 firstly among patients with ADLH < 2 vs ≥2 and secondly among patients with CPS < 2 vs ≥2. Among patients with both FI-PAC ≥0.40 and ALDH ≥2, the odds ratio for prolonged hospital stay was greater than that of sole ADL deficit (ADLH+FI-PAC OR [95% CI] 7.49 [5.47─10.26], sole ADL deficit 3.35 [2.40–4.68]). The situation was the same for CPS (CPS + FI-PAC 5.45 [4.05─7.33], sole CPS deficit 1.71 [1.24─2.36]). For other outcomes, no such differences were observed.

## Discussion

In this large retrospective cohort study of older patients in a post-acute care setting, we derived a Frailty Index (FI-PAC) from the interRAI Post-Acute Care instrument (interRAI-PAC) to summarize the results of the comprehensive assessment. A Frailty Index has previously been derived from the interRAI Acute Care instrument [25], and it has been shown to predict multiple adverse outcomes in hospitalized older patients [10], but the interRAI-PAC has not been previously used for that purpose. Most variables are the same in the FI-PAC as in the Frailty Index derived from the interRAI assessment system for Acute Care (FI-AC), but one difference is that instead of using single variables, we included the Depression Rating Scale (DRS), Pain Scale (PAIN), and Aggressive Behaviour Scale (ABS) in the FI-PAC. Another difference is that we did not include the number of medications in the FI-PAC. In addition, we included walking speed.

We succeeded in deriving a Frailty Index from the interRAI-PAC with the expected normal distribution in this study population [25, 39]. The distribution of the Frailty Index is usually skewed in population-based samples, but it tends to change to a normal distribution in more morbid and unwell groups of older people [41]. However, a skewed distribution was also found in hospitalized older patients in a study by Cesari et al. [11]. This discrepancy could be attributed to the better functional ability of the patients in their study. The mean score for the FI-PAC was 0.34, which was close to the mean score of 0.32 for the FI-AC [25]. There were no significant differences between age and sex groups, and this finding is consistent with the finding of Hubbard et al. [25].

It transpired that the FI-PAC was associated with both prolonged hospital stay and in-hospital mortality, and it had a good discriminative ability (both AUCs over 0.70). Previous studies have not dealt with length of hospital stay in the post-acute care setting, but the results from acute care showed an association between the Frailty Index and prolonged length of stay [8, 9]. In accordance with our results, Hubbard et al. found an association between the FI-AC and in-hospital mortality [10]. This finding is also consistent with previous studies that have examined the predictive ability of the Frailty Index [11] and the Clinical Frailty Scale [40, 41] for in-hospital mortality in the acute care setting.

It was noted also that the FI-PAC associated with emergency department admission, but the predictive ability was only modest. This result may be explained by the fact that most short-term readmissions to acute care hospitals are due to medical issues [42, 43] – for example, acute and chronic diseases – and the impact of these diseases on admission to acute care is greater than that of frailty status.

Interestingly, the FI-PAC was equal but not superior to ADLH in predicting prolonged hospital stay and in-hospital mortality. However, having a high Frailty Index significantly increased the odds for adverse hospital outcomes in patients with ADL impairments or cognitive decline compared to the effects of these conditions alone. In their analysis based on the FI-AC, Hubbard et al. did not compare the predictive ability of the FI-AC to the standard interRAI scales [10]. Although several studies have shown that ADL impairment upon admission to acute hospital is a strong predictor of prolonged hospital stay and mortality in older patients [14, 43, 45], it was surprising that functional impairment, measured by the short ADLH scale, was as good a prognostic instrument as the multicomponent Frailty Index. These results are, however, in agreement with Chen’s findings, which showed that frailty and functional dependence were comparable in predicting short-term outcomes after gastrointestinal surgery [46]. A possible explanation might be that frailty is a complex phenomenon and different instruments – for example, the Frailty Index – can measure only some aspects of it [3]. Although the Frailty Index consists of a variety of different health-related items, it more or less represents a sum of comorbidities and disabilities rather than a measure of the biological aspects of frailty [47]. If measuring biological (phenotypic) frailty had been possible in our study, the results might be different.

It can thus be suggested that, in clinical practice, calculating the Frailty Index for the purpose of identifying patients with poor outcomes does not bring additional value over assessment of functional ability. Instead, the detection of functional impairment can be used to define frailty [48]. From a clinical point of view, assessment of the patient’s functional ability is simple, quick, and inexpensive, and it is usually already part of the nurses’ assessment protocol. Owing to the multifactorial basis of functional impairment [49], factors underlying each person’s functional decline are probably different regardless of similar scores on the Frailty Index. Thus, the detection of functional impairment should in turn lead to the comprehensive clinical and interprofessional evaluation of the patient in order to clarify underlying factors and make a plan for proper treatment and rehabilitation.

For clinical decision making, cut-off points with approximate discrimination between robust, prefrail and frail individuals have been developed. In older adults with functional decline, the cut-off point is about 0.25 between robust and prefrail and about 0.40 between prefrail and frail [10, 38]. We considered it important to clarify the clinically relevant cut-off points for the FI-PAC that can be used to differentiate persons who are likely to experience adverse outcomes during their hospitalization from those who are likely to survive without complications. Optimal cut-off points, based on the ROC curves, varied from 0.30 to 0.35 in our study population. The problem with the Frailty Index in this patient population is that by using the cut-off point of 0.35, half of the patients are classified as being at risk for adverse outcomes. However, scores that were lower than the cut-off points ruled out most patients who did not face adverse outcomes during hospitalization.

The strengths of our study are the representative sample size and quite homogenous patient population, the complete records, and the representation of real-life patients due to the retrospective nature of the study. However, a note of caution is due here since our materials did not include all patients that had a treatment period in a post-acute care hospital during the study period, because the interRAI assessment was not made for everybody. There are many possible reasons for missing assessments. One reason is that the introduction of interRAI-PAC was gradual in different wards, but hospital discharge records were collected the same period of time from both hospitals. In addition, the assessment was not done for the patients who were in a terminal care phase and to the patients with suspected hospital stay for less than seven days. Another reason may be related to the fact that the completion of an interRAI assessment is time and resource demanding [50], which may lead to a substantial number of the missing assessments in real-life clinical context [51]. However, this is unlikely to cause systematic bias in our analysis.

Another source of uncertainty is our lack of knowledge of incidents occurring during the whole hospital treatment period of the patient – for example, the length of stay in an acute care hospital, diagnoses of acute diseases, or treatments given. The predictive ability of the FI-PAC probably varies between different patient groups, for instance between patients whose reason for hospitalization is acute disease versus patients whose reason for the hospital stay is postoperative rehabilitation. Therefore, caution must be applied when applying our results to diverse patient groups. In addition, although our materials cover all post-acute care in our city and although the patients represent unselected population (in terms of social or insurance status), it is acknowledged that in international context, the current patient numbers are modest and the results may not be fully generalizable to other health care systems.

## Conclusions

It is possible to derive Frailty Index from the interRAI-PAC and such FI predicts adverse hospital outcomes as expected. However, its predictive ability was not better than that of the ADLH scale and because most patients had FI values predictive of adverse outcomes, FI-PAC does not seem to aid in decision-making at the level of an individual patient. In clinical practice, the assessment of functional ability is an important and simple way to assess the patient’s prognosis. Patients with functional impairment should be evaluated carefully in order to clarify underlying factors and make a plan for treatment and rehabilitation. Future research should focus on the comparison of the phenotypic (biological) frailty model and the Frailty Index in predicting hospital outcomes.

## Supplementary information

**Additional file 1.** The Frailty Index derived from interRAI Post-Acute Care instrument. Table that describes the formation of Frailty Index from interRAI Post-Acute Care instrument.

## Data Availability

The datasets generated and analysed during the current study are not publicly available, because they represent confidential health information of the included patients, and distribution of such data is forbidden according to data protection legislation. Furthermore, the authorities responsible for the health data have granted access to these data only for the present study. Summarized data is available on reasonable request from the corresponding author.

## Abbreviations

ABS: Aggressive Behaviour Scale
ADLH: Activities of Daily Living Hierarchy Scale
ADLs: Activities of daily living
AUC: Area under the ROC curve
BALD: Basic activities of daily living
BMI: Body mass index
CGA: Comprehensive Geriatric Assessment
CHESS: Changes in Health, End-stage disease, and Signs and Symptoms Scale
CIs: Confidence intervals
CPS: Cognitive Performance Scale
DRS: Depression Rating Scale
FI-AC: Frailty Index derived from the interRAI assessment system for Acute Care
FI-PAC: Frailty Index derived from the interRAI assessment system for Post-Acute Care
interRAI-PAC: interRAI assessment system for Post-Acute Care
NPV: Negative predictive value
PAIN: Pain Scale
PPV: Positive predictive value
ROC: Receiver operating characteristic curve
