# Supplementary material for: Frailty Index and functional level upon admission predict hospital outcomes: an interRAI-based cohort study of older patients in post-acute care hospitals
Source: BMC Geriatr. 2020 May 5;20:160. doi: 10.1186/s12877-020-01550-7 (PMC7201739; doi:10.1186/s12877-020-01550-7)
Supplement: Supplementary file 1 — Additional file 1. The Frailty Index derived from interRAI Post-Acute Care instrument. Table that describes the formation of Frailty Index from interRAI Post-Acute Care instrument. [file 12877_2020_1550_MOESM1_ESM.pdf]

Additional file 1. Frailty Index Post-Acute Care

	icode	Question	Value Labels	InterRAI Cut Offs	FI score	Running Denominator
C. Cognition	iC1	Cognitive skills for daily decision making	0 Independent<br>1 Modified independence<br>2 Minimally impaired<br>3 Moderately impaired<br>4 Severely impaired<br>5 No discernible consciousness, coma	0<br>1 – 2<br>3 - 5	0<br>0.5<br>1	1
	iC2a	Short term memory	0 Memory OK<br>1 Memory problem	0<br>1	0<br>1	2
	iC2b	Long term memory	0 Memory OK<br>1 Memory problem	0<br>1	0<br>1	3
	iC2c	Procedural memory	0 Memory OK<br>1 Memory problem	0<br>1	0<br>1	4
	iC2d	Situational memory	0 Memory OK<br>1 Memory problem	0<br>1	0<br>1	5
	iC3a	Easily distracted	0 Behaviour not present<br>1 Behaviour present, consistent with usual functioning<br>2 Behaviour present, different from usual functioning	0<br>1 - 2	0<br>1	6
	iC3b	Disorganised speech	0 Behaviour not present<br>1 Behaviour present, consistent with usual functioning<br>2 Behaviour present, different from usual functioning	0<br>1 - 2	0<br>1	7
	iC3c	Mental function varies over course of day	0 Behaviour not present<br>1 Behaviour present, consistent with usual functioning<br>2 Behaviour present, different from usual functioning	0<br>1 - 2	0<br>1	8
	iC4	Acute change in mental status	0 No<br>1 Yes	0<br>1	0<br>1	9
D. Communication and Vision	iD3	Hearing	0 Adequate<br>1 Minimal difficulty<br>2 Moderate difficulty<br>3 Severe difficulty<br>4 No hearing	0<br>1<br>2 - 4	0<br>0.5<br>1	10
	iD5	Vision	0 Adequate<br>1 Minimal difficulty<br>2 Moderate difficulty<br>3 Severe difficulty<br>4 No vision	0<br>1<br>2 - 4	0<br>0.5<br>1	11

	icode	Question	Value Labels	InterRAI Cut Offs	FI score	Running Denominator
E. Mood and Behaviour	iE1a-g	Indicators of depression, anxiety or sad mood<br><br>Depression Rating Scale (0-14)	Calculate the Depression Rating Scale	DRS 0<br>DRS 1-2<br>DRS ≥3	0<br>0.5<br>1	12
	iE3b-d,f	Indicators of behaviour symptoms<br><br>Aggressive Behaviour Scale (0-12)	Calculate the Aggressive Behaviour Scale	ABS 0<br>ABS 1-3<br>ABS ≥4	0<br>0.5<br>1	13
F. Functional status	iF1a	Bathing	0 Independent<br>1 Independent, setup help only<br>2 Supervision- oversight, cueing<br>3 Limited assistance- guided manoeuvring of limbs<br>4 Extensive assistance- 1 person weight bearing support<br>5 Maximal assistance- 2 person weight bearing support<br>6 Total dependence<br>8 Activity did not occur	0 - 1<br><br>2<br><br>3 – 6, 8	0<br><br>0.5<br><br>1	14
	iF1b	Personal hygiene	0 Independent<br>1 Independent, setup help only<br>2 Supervision- oversight, cueing<br>3 Limited assistance- guided manoeuvring of limbs<br>4 Extensive assistance- 1 person weight bearing support<br>5 Maximal assistance- 2 person weight bearing support<br>6 Total dependence<br>8 Activity did not occur	0–1<br><br>2<br><br>3 – 6, 8	0<br><br>0.5<br><br>1	15
	iF1c	Dressing upper body	0 Independent<br>1 Independent, setup help only<br>2 Supervision- oversight, cueing<br>3 Limited assistance- guided manoeuvring of limbs<br>4 Extensive assistance- 1 person weight bearing support<br>5 Maximal assistance- 2 person weight bearing support<br>6 Total dependence<br>8 Activity did not occur	0 - 1<br><br>2<br><br>3 – 6, 8	0<br><br>0.5<br><br>1	16
	iF1d	Dressing lower body	0 Independent<br>1 Independent, setup help only<br>2 Supervision- oversight, cueing<br>3 Limited assistance- guided manoeuvring of limbs<br>4 Extensive assistance- 1 person weight bearing support<br>5 Maximal assistance- 2 person weight bearing support<br>6 Total dependence<br>8 Activity did not occur	0 - 1<br><br>2<br><br>3 – 6, 8	0<br><br>0.5<br><br>1	17

	icode	Question	Value Labels	InterRAI Cut Offs	FI score	Running Denominator
G. Continence	iG1	Bladder continence	0 Continent<br>1 Control with catheter or ostomy<br>2 Infrequently incontinent<br>3 Occasionally incontinent<br>4 Frequently incontinent<br>5 Incontinent<br>8 Did not occur – no urine output in last 3 days	0 – 1<br><br>2 – 3<br><br>4,5,8	0<br><br>0.5<br><br>1	33
	iF1g	Toilet Transfers	0 Independent<br>1 Independent, setup help only<br>2 Supervision- oversight, cueing<br>3 Limited assistance- guided manoeuvring of limbs<br>4 Extensive assistance- 1 person weight bearing support<br>5 Maximal assistance- 2 person weight bearing support<br>6 Total dependence<br>8 Activity did not occur	0 – 1<br><br>2<br><br>3 – 6, 8	0<br><br>0.5<br><br>1	19
	iF1h	Toilet use	0 Independent<br>1 Independent, setup help only<br>2 Supervision- oversight, cueing<br>3 Limited assistance- guided manoeuvring of limbs<br>4 Extensive assistance- 1 person weight bearing support<br>5 Maximal assistance- 2 person weight bearing support<br>6 Total dependence<br>8 Activity did not occur	0 – 1<br><br>2<br><br>3 – 6, 8	0<br><br>0.5<br><br>1	20
	iF1i	Bed mobility	0 Independent<br>1 Independent, setup help only<br>2 Supervision- oversight, cueing<br>3 Limited assistance- guided manoeuvring of limbs<br>4 Extensive assistance- 1 person weight bearing support<br>5 Maximal assistance- 2 person weight bearing support<br>6 Total dependence<br>8 Activity did not occur	0 – 1<br><br>2<br><br>3 – 6, 8	0<br><br>0.5<br><br>1	21
	iF1j	Eating	0 Independent<br>1 Independent, setup help only<br>2 Supervision- oversight, cueing<br>3 Limited assistance- guided manoeuvring of limbs<br>4 Extensive assistance- 1 person weight bearing support<br>5 Maximal assistance- 2 person weight bearing support<br>6 Total dependence<br>8 Activity did not occur	0 – 1<br><br>2<br><br>3 – 6, 8	0<br><br>0.5<br><br>1	22

	icode	Question	Value Labels	InterRAI Cut Offs	FI score	Running Denominator
	iF2a	Mode of locomotion	0 Walking, no assistive device<br>1 Walking, uses assistive device<br>2 Wheelchair, scooter<br>3 Bedbound	0<br>1<br>2 - 3	0<br>0.5<br>1	23
	iF2b	Walking speed	Count Timed 4-meter walk distance (m)/ walking time (s)	>= 0,8 m/s<br>< 0,8 m/s<br>Not performed	0<br>1<br>1	24
	iG1	Bladder continence	0 Continent<br>1 Control with catheter or ostomy<br>2 Infrequently incontinent<br>3 Occasionally incontinent<br>4 Frequently incontinent<br>5 Incontinent<br>8 Did not occur – no urine output in last 3 days	0 – 1<br><br>2 – 3<br><br>4,5,8	0<br><br>0.5<br><br>1	25
	iG4	Bowel continence	0 Continent<br>1 Control with ostomy<br>2 Infrequently incontinent<br>3 Occasionally incontinent<br>4 Frequently incontinent<br>5 Incontinent<br>8 Did not occur – no bowel movement in last 3 days	0 – 1<br><br>2 – 3<br><br>4,5,8	0<br><br>0.5<br><br>1	26
H. Diagnoses	iH1a	Hip fracture during last 30 days	0 Not present<br>1 Primary diagnosis of current stay<br>2 Diagnosis present, receiving active treatment<br>3 Diagnosis present, monitored but no active treatment	0<br>1-3	0<br>1	27
	iH1b	Other fracture during last 30 days	0 Not present<br>1 Primary diagnosis of current stay<br>2 Diagnosis present, receiving active treatment<br>3 Diagnosis present, monitored but no active treatment	0<br>1-3	0<br>1	28
	iH1c	Alzheimers disease	0 Not present<br>1 Primary diagnosis of current stay<br>2 Diagnosis present, receiving active treatment<br>3 Diagnosis present, monitored but no active treatment	0<br>1-3	0<br>1	29
	iH1d	Dementia other than ALZ	0 Not present<br>1 Primary diagnosis of current stay<br>2 Diagnosis present, receiving active treatment<br>3 Diagnosis present, monitored but no active treatment	0<br>1-3	0<br>1	30

	icode	Question	Value Labels	InterRAI Cut Offs	FI score	
	iH1h	Parkinson´s disease	0 Not present<br>1 Primary diagnosis of current stay<br>2 Diagnosis present, receiving active treatment<br>3 Diagnosis present, monitored but no active treatment	0<br>1-3	0<br>1	31
	iH1j	Stroke/ SVA	0 Not present<br>1 Primary diagnosis of current stay<br>2 Diagnosis present, receiving active treatment<br>3 Diagnosis present, monitored but no active treatment	0<br>1-3	0<br>1	32
	iH1k	Coronary heart disease	0 Not present<br>1 Primary diagnosis of current stay<br>2 Diagnosis present, receiving active treatment<br>3 Diagnosis present, monitored but no active treatment	0<br>1-3	0<br>1	33
	iH1l	Congestive heart failure	0 Not present<br>1 Primary diagnosis of current stay<br>2 Diagnosis present, receiving active treatment<br>3 Diagnosis present, monitored but no active treatment	0<br>1-3	0<br>1	34
	iH1m	COPD	0 Not present<br>1 Primary diagnosis of current stay<br>2 Diagnosis present, receiving active treatment<br>3 Diagnosis present, monitored but no active treatment	0<br>1-3	0<br>1	35
	iH1n	Anxiety	0 Not present<br>1 Primary diagnosis of current stay<br>2 Diagnosis present, receiving active treatment<br>3 Diagnosis present, monitored but no active treatment	0<br>1-3	0<br>1	36
	iH1p	Depression	0 Not present<br>1 Primary diagnosis of current stay<br>2 Diagnosis present, receiving active treatment<br>3 Diagnosis present, monitored but no active treatment	0<br>1-3	0<br>1	37
	iH1t	Cancer	0 Not present<br>1 Primary diagnosis of current stay<br>2 Diagnosis present, receiving active treatment<br>3 Diagnosis present, monitored but no active treatment	0<br>1-3	0<br>1	38
	iH1u	Diabetes	0 Not present<br>1 Primary diagnosis of current stay<br>2 Diagnosis present, receiving active treatment<br>3 Diagnosis present, monitored but no active treatment	0<br>1-3	0<br>1	39
I. Health Conditions	iI1	Falls	0 No fall in last 90 days<br>1 No fall in last 30 days but fell 31-90 days ago<br>2 One fall in last 30 days<br>3 Two or more falls in last 30 days	0<br>1<br>2, 3	0<br>0.5<br>1	40

	icode	Question	Value Labels	InterRAI Cut Offs	FI score	Running Denominator
	iI3a	Balance: Difficult to move self to standing	0 Not present<br>1 Present but not exhibited last 3 days<br>2 Exhibited on 1 of last 3 days<br>3 Exhibited on 2 of last 3 days<br>4 Exhibited daily in last 3 days	0<br><br>1-4	0<br><br>1	41
	iI3b	Balance: Difficult to turn self around	0 Not present<br>1 Present but not exhibited last 3 days<br>2 Exhibited on 1 of last 3 days<br>3 Exhibited on 2 of last 3 days<br>4 Exhibited daily in last 3 days	0<br><br>1-4	0<br><br>1	42
	iI3c	Dizziness	0 Not present<br>1 Present but not exhibited last 3 days<br>2 Exhibited on 1 of last 3 days<br>3 Exhibited on 2 of last 3 days<br>4 Exhibited daily in last 3 days	0<br><br>1-4	0<br><br>1	43
	iI3d	Unsteady gait	0 Not present<br>1 Present but not exhibited last 3 days<br>2 Exhibited on 1 of last 3 days<br>3 Exhibited on 2 of last 3 days<br>4 Exhibited daily in last 3 days	0<br><br>1-4	0<br><br>1	44
	i3l	Constipation	0 Not present<br>1 Present but not exhibited last 3 days<br>2 Exhibited on 1 of last 3 days<br>3 Exhibited on 2 of last 3 days<br>4 Exhibited daily in last 3 days	0<br><br>1-4	0<br><br>1	45
	iI3o	Difficulty falling asleep	0 Not present<br>1 Present but not exhibited last 3 days<br>2 Exhibited on 1 of last 3 days<br>3 Exhibited on 2 of last 3 days<br>4 Exhibited daily in last 3 days	0<br><br>1-4	0<br><br>1	46
	iI4	Dyspnoea	0 Absence of symptom<br>1 Absent at rest, but present with moderate activity<br>2 Absent at rest, but present with normal activity<br>3 Present at rest	0<br><br>1-3	0<br><br>1	47
	iI5	Fatigue- inability to complete normal activity	0 None<br>1 Minimal<br>2 Moderate<br>3 Severe<br>4 Unable to commence any day-to-day activity	0<br><br>1<br><br>2 - 4	0<br><br>0.5<br><br>1	48

	icode	Question	Value Labels	InterRAI Cut Offs	FI score	Running Denominator
	i6a-b	Indicators of pain<br><br>PAIN-scale (0-4)	Calculate the PAIN-scale	PAIN 0<br>PAIN 1<br>PAIN 2-4	0<br>0.5<br>1	49
	iI7c	End-stage disease	0 No<br>1 Yes	0<br>1	0<br>1	50
J. Oral and Nutritional Status	iJ1ab, iJ1bb	Calculate BMI	BMI	20 - 30<br>< 20 or >30	0<br>1	51
	iJ2a	Weight loss of 5% or more in last 30 days, 10% or more in last 180 days	0 No<br>1 Yes	0<br>1	0<br>1	52
	iJ2b	Dehydrated	0 No<br>1 Yes	0<br>1	0<br>1	53
	iJ3	Mode of intake	0 Normal<br>1 Modified independent –limited solid food<br>2 Requires diet modification to swallow solid food<br>3 Requires diet modification to swallow liquids<br>4 Can swallow only pureed solids and thickened fluids<br>5 Combined oral and parenteral or tube feeding<br>6 Nasogastric tube feeding only<br>7 Abdominal feeding tube e.g. PEG<br>8. Parenteral feeding only<br>9 Activity did not occur	0<br>1-2<br>3 - 9	0<br>0.5<br>1	54
K. Skin	iK1-iK2	Pressure ulcer-current or prior	0 No<br>1 Yes	0<br>1	0<br>1	55
	iK3-iK6	Skin problems (any of presence of skin ulcer, major skin problem, skin tears, other skin conditions)	0<br>≥1	0<br>1	0<br>1	56
	iK7	Foot problems	0 No foot problems<br>1 Foot problems, no limitation in walking<br>2 Foot problems limit walking<br>3 Foot problems prevent walking<br>4 Foot problems, does not walk for other reasons	0<br>1-4	0<br>1	57

The denominator from PAC ITEMS is 57. For complete interRAI PAC forms, the individual's FI is calculated by dividing their number of deficits by 57. The denominator can be adjusted for missing items.
